# Chicago Medical College

**Published:** 1879-05

**Authors:** 


The Faculty of the Chicago Medical College announce that Professor EDWARD W. JENKS, M. D., of the Detroit Medical College, has accepted an appointment to the Chair of Medical and Surgical Diseases of Women and Clinical Gynecology. Dr. Jenks has already acquired a national reputation so prominent, in this department, as a teacher and operator, that it is unnecessary to speak of the advantages which are thus secured to the College. Dr. Jenks is to change his residence and field of labor to that city at an early day.

